# Urban Ecological Security Simulation and Prediction Using an Improved Cellular Automata (CA) Approach—A Case Study for the City of Wuhan in China

**DOI:** 10.3390/ijerph14060643

**Published:** 2017-06-15

**Authors:** Yuan Gao, Chuanrong Zhang, Qingsong He, Yaolin Liu

**Affiliations:** 1 School of Resource and Environmental Science, Wuhan University, 129 Luoyu Road, Wuhan 430079, China; 2015102050045@whu.edu.cn; 2 Department of Geography and Center for Environmental Sciences and Engineering, University of Connecticut, Storrs, CT 06269-4148, USA; cindy.zhang@uconn.edu; 3 Key Laboratory of Geographic Information System, Ministry of Education, Wuhan University, 129 Luoyu Road, Wuhan 430079, China; 4 Collaborative Innovation Center for Geospatial Information Technology, Wuhan 430079, China

**Keywords:** urban ecological security, simulation and prediction, pressure-state-response (PSR), cellular automata (CA), geographically weighted regression (GWA)

## Abstract

Ecological security is an important research topic, especially urban ecological security. As highly populated eco-systems, cities always have more fragile ecological environments. However, most of the research on urban ecological security in literature has focused on evaluating current or past status of the ecological environment. Very little literature has carried out simulation or prediction of future ecological security. In addition, there is even less literature exploring the urban ecological environment at a fine scale. To fill-in the literature gap, in this study we simulated and predicted urban ecological security at a fine scale (district level) using an improved Cellular Automata (CA) approach. First we used the pressure-state-response (PSR) method based on grid-scale data to evaluate urban ecological security. Then, based on the evaluation results, we imported the geographically weighted regression (GWR) concept into the CA model to simulate and predict urban ecological security. We applied the improved CA approach in a case study—simulating and predicting urban ecological security for the city of Wuhan in Central China. By comparing the simulated ecological security values from 2010 using the improved CA model to the actual ecological security values of 2010, we got a relatively high value of the kappa coefficient, which indicates that this CA model can simulate or predict well future development of ecological security in Wuhan. Based on the prediction results for 2020, we made some policy recommendations for each district in Wuhan.

## 1. Introduction

Ecological security refers to a non-threatened state [1,2,3], which may include the state of human life, health, well-being, basic rights, livelihood resources, necessary resources, social order, and human ability to adapt to environmental changes. Ecological security reflects the degree of ecosystem integrity and health [1], and it may provide guarantee for sustainability of an eco-economic system [4]. Ecological security research began in the last century [5,6,7]. Important literature on this concept includes the “Ecological risk assessment outline” published by the United States Environmental Protection [8] and the “Global vulnerability assessment framework for environmental risk” proposed by Clark et al. [9]. Ecological security is a concept opposite to ecological risk. Ecological security measures the safeguard of ecological systems while ecological risk assesses the danger or destruction of ecological systems. However ecological security and ecological risk are related. In fact, parts of the concept of ecological security come from ecological risk [10,11,12]. Because many ecological disasters or degradation are caused by human activities, measurement of ecological security should represent the capability of human activities. Ecological security may be assessed using an indicator system for environmental pressures or degradation.

Urban ecological security is an important research topic [13]. The urban area, as the center of socio-economic development, is a highly populated ecological system and is more fragile in terms of ecological security [2,4,14]. It is, therefore, imperative to study urban ecological security. In addition, studying urban ecological security may benefit for carrying out efficient environmental management [15]. With increasing concerns over urban ecological security, many methods have been proposed in the literature for assessing urban ecological security. These methods mainly include mathematical models, system dynamics methods, ecological footprint methods [16], and landscape ecology methods [15,17,18]. The commonly used mathematical model methods include the pressure-state-response (PSR) model, the comprehensive index method [19], the analytic hierarchy process method [20,21], the gray correlation degree method [22], the matter element evaluation method, and the principal component projection method. However, some of these methods, such as the comprehensive index methods and ecological footprint methods have limitations in the study of urban ecological security. These methods may be good at evaluating the status of an administrative region, but they cannot reflect the dynamic process of the ecological security change, especially at a fine scale. Among these mathematical methods, the PSR model for human and environmental systems [23,24,25,26], which was jointly developed by the United Nations Economic Cooperation and Development (OECD) and the United Nations Environment Program (UNEP), and the comprehensive evaluation index system established by U.S. Environmental Protection Agency (USEPA) [27] are particularly important.

However, most of the research on urban ecological security using the aforementioned methods focused on evaluating current or past status of the ecological environment. Very few studies in the literature have been carried out to simulate or predict future changes in urban ecological security. Nevertheless, predicting future changes in urban ecological security is important [28,29]. The predicted change of urban ecological security could be used to make reasonable environmental policies for urban sustainability development [30,31,32]. The predicted change of urban ecological security could also be useful for urban planners to develop more realistic urban development plans. However, there is a lack of literature in simulating or predicting future changes of urban ecological security. Based on our best knowledge, we only found a couple of publications in literature for forecasting urban ecological security. For example, Gong et al. [28] and Yang et al. [29] forecasted urban ecological security using the classical cellular automata (CA) models. However, the classical CA models consider only short-range spatial dependence and do not consider long-range spatial dependence (e.g., not immediately connected neighbor cells but located far away cells) [33]. Therefore, it is difficult to make an accurate simulation or prediction of the dynamic urban ecological security changes among the neighborhoods.

In addition, there is little literature exploring the urban ecological environment at a fine scale. In most existing literature, urban ecological security was studied at coarse scales (global, regional, city, or county levels) [34,35]. In those studies, a whole city or a county was given only one security value [21,36]. It is, therefore, impossible to figure out how things are going on inside the city [33]. Nevertheless, urban areas are heterogeneous [37]. Urban ecological security may be different at different locations in the fine scales such as district level. It is, therefore, improper to treat the city as homogeneous and give the whole city one urban ecological security value. One security value cannot represent the uneven status of ecological security inside a city. Different districts or neighborhoods in a city may have different ecological security values. Therefore, we need to simulate urban ecological security values at a fine scale to give us more information about the heterogeneity of a city. This may be used for better ecological understanding of integrated human-natural urban systems and undertake wise urban planning. Compared to the coarse scales such as city scale, a fine scale could help to find out the uneven status of ecological security inside a city and could give more information about the heterogeneous ecological security inside a city.

To overcome these limitations and fill-in the literature gaps, in this study, we simulated and predicted urban ecological security at a fine scale (district level) using an improved CA approach to better understand spatial heterogeneity of urban ecosystems. There are two major new contributions of the improved CA approach. First, we used the pressure-state-response (PSR) method based on grid-scale data to evaluate urban ecological security. We considered urbanization as the most significant driving force causing ecological and environmental problems. We carefully quantified the ecological status of the urbanization areas, because the biggest impetus to the ecological security problem is the increased urbanization processes. To better quantify urban ecological security, we used three dimensions to characterize urban growth. The three dimensions include urban growth degree (UGD), urban growth capacity (UGC), and urban growth pattern (UGP). We evaluated ecological security states from the view of environmental structure, ecological function value, and resilience of the ecosystem.

Second, in simulating and predicting the urban ecological security process, we extended the classical CA model by introducing the geographic weighting regression (GWR) concept into the transformation rules of the classical CA model. GWR can take into account spatial variation of the sampling unit [38], therefore, it may make simulation of the dynamic urban ecological security changes among different neighborhoods more accurate.

We applied the improved CA approach in a case study—simulating and predicting urban ecological security for the city of Wuhan of Central China. Through the case study, we aimed to verify the potential of the improved CA method for more accurately simulating and predicting urban ecological security.

## 2. Materials and Methods

### 2.1. Study Area

**Figure 1:**
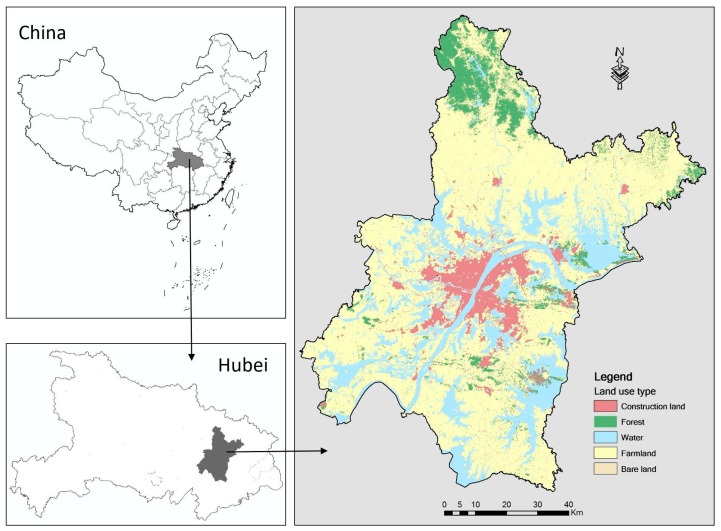
Location of the study area.

The city of Wuhan is one of the fastest growing cities in central China, located in the middle reaches of the Yangtze River (see Figure 1). It is the capital of Hubei Province. Wuhan city constitutes seven districts and has a total area of 8549 km^2^, of which 39.27% consists of plains and 18.17% is hilly and mountainous regions. Wuhan has experienced rapid urbanization and a significant economic growth since last century. From 2005 to 2010, the urban area expanded to 552.61 km^2^ (Wuhan Municipal Bureau of Statistics 2005, 2010). Its population grew from 8.58 million to 10.61 million and its economy expanded from 226.12 billion to 1090.56 billion yuan. The city has been witnessed an obvious and rapid urbanization.

### 2.2. Data Sources

Land use data were mainly derived from remotely sensed images. The remotely sensed images include two multi-spectral Landsat MSS/TM/ETM+ images in the years 2005 and 2010. The resolution of the Landsat MSS/TM/ETM+ images is 30 m. The images were downloaded from the U.S. Geological Survey (USGS). To ensure accuracy of the image classification results, all the selected images are cloudless and with thick vegetation coverage. Before classification, all the images and other Geographic Information System (GIS) data were geometrically rectified to the World Geodetic System-1984 Coordinate System (WGS-84) and the Universal Transverse Mercator coordinate system. In total, 90 ground control points were selected as supplementary data. We did the classification using the maximum likelihood method. According to natural ecological and land use attributes, land uses/covers from remotely sensed images were always classified into six types. However, grassland has a very small proportion in Wuhan. The images were, therefore, classified into other five types of land use/cover, namely construction land, forest, farmland, water, and bare land. Meanwhile, we randomly selected 200 training samples with sufficient information on the spectral characteristics for each land use type. In addition, we selected 100 field points for checking classification accuracy. The overall classification accuracy values for the images are from 81.41% to 88.00%, and the kappa coefficient values are from 0.77 to 0.85, which indicate a high classification accuracy [39,40]. Based on the classification results, the proportions of construction land, forest, farmland, water, and bare land in Wuhan in 2010 are 7.78%, 8.34%, 61.99%, 21.40%, and 0.49%, respectively. Population data were collected from the fifth census and the sixth census in China at the street level. Socio-economic data mainly include national and regional planning documents and policies, which were downloaded from the government website [41].

### 2.3. Methods

#### 2.3.1. The Flow Chart of the Methods

**Figure 2:**
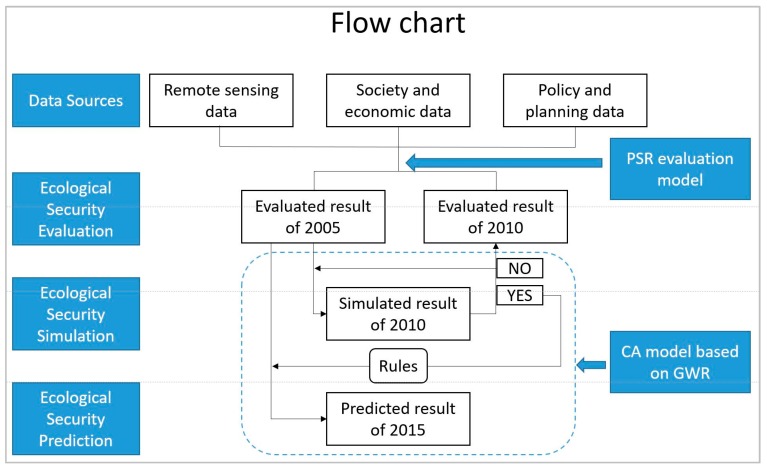
The flow chart of the methods. CA: cellular automata; PSR: pressure-state-response.

The methods include two main components: evaluation of urban ecological security using the PSR model and simulation and prediction of ecological security using the CA model with the GWR concept. Figure 2 illustrates the flow chart of the methods. The procedure of the methods is introduced as follows:

Step 1: We evaluated the ecological security classes of 2005 and 2010 using the PSR model.
Step 2: We used the data of 2005 to conduct a five-year simulation based on the CA rules. We obtained the simulated result of 2010 based on the five-year simulation.
Step 3: We compared the simulated result of 2010 and the evaluated result of 2010 to verify the simulation accuracy in the second step.
Step 4: We repeated step 2 and step 3 until a satisfactory level of accuracy was obtained in step 3. Then we recorded the CA rules of the simulation. The total number of the iterations of the 5-year simulation was recorded as *N*.
Step 5: We then obtained the predicted result for 2020, which is a 15-year simulation, by using the rules obtained in step 4. We took *3N* as the total number of iterations in this 15-year simulation.

#### 2.3.2. PSR Evaluation Model

PSR model is a comprehensive evaluation model for environmental problems proposed by the United Nations Economic Cooperation and Development (OECD). It is composed of the pressure index for ecological environment problems, state index for eco-environment status, and response index for social and economic measures that quantify human’s response to ecological problems. The pressure index consists of three indicators: urban growth degree (UGD) [29], urban growth capacity (UGC) [42,43,44,45,46,47,48], and urban growth pattern (UGP) [49,50,51]. The ecological security status of an ecosystem was considered from three aspects: ecological function, ecological structure, and ecological resilience [52]. In order to make the research scale more refined and better reflect ecological state of the administrative units, the indicators of these three aspects were chosen at landscape scale [53]. The landscape ecological function (LEF) was assessed mainly based on the ecological service values. The most frequently used method proposed by Costanza et al. [54] and Xie et al. [55] was used to measure the ecological service values for different land use types in this study. The landscape ecological structure (LES) was measured using (1) Shannon’s diversity index (SHDI), which measures the number of different land cover classes present; (2) the area-weighted plots fractal dimension index that reflects shape complexity across a range of spatial scales; and (3) modified Simpson’s evenness index, which ensures an even distribution of areas among patch types [51].

Landscape ecological resilience (LER) is an important factor for sustainability of regional ecology. LER measures the disturbance that an ecosystem can bear until its characteristics change [56]. In this study, we used the fragility index to measure this disturbance. It is calculated by the inverse of number of patches per unit area (PD). The 8-way neighbors method was used to define patches that can well represent the ecological resilience of a region [52].

The ecological security responses were interpreted by policy measures such as ecological zoning and environmental plans. From 2005 to 2010, the specific ecological responses of Wuhan City included “ecological bottom line”, “ecological development area”, “prospective ecological area”, “tricyclic rectification area”, and “urban concentrated construction area”.

All of the aforementioned landscape indices were calculated using the software Fragstats [57,58]. Delphi method was used to assign the weight for each index. Compared to the objective weight allocation methods, Delphi method can better reflect the regional differences, and is more in line with the objective of this study so this method was employed. Considering the mechanism of the PSR model and the richness of the data, we assigned the state a weight of 0.5, which can better display the ecological security of Wuhan city. Since both the UGD and UGC in the pressure part and LEF and LES in the state part played important roles in the evaluation of urban ecological security, they were assigned higher weights. Table 1 lists the specific PSR evaluation indices and their weights.

Before performing the evaluation, the original data of different dimensions were standardized. The extremum standardization method was used for this purpose.

#### 2.3.3. GWR-Based Cellular Automata (CA) Model

Cellular automata (CA) was a commonly used model for simulating and predicting urban growth [42,58,59]. CA models can take into account dynamic changes of a city caused by complex factors such as nature and socio-economy, and use simple rules to simulate the complicated temporal and spatial dynamic processes [60,61,62]. In addition, it has the advantage of easily integrating itself with high-resolution remotely sensed images and geographic information systems [63]. This makes it popular for simulating urban dynamic changes. Like general urban dynamic change processes, urban ecological security could be described as a synthesis of components of coupled human and natural ecosystems. Urban ecological security exhibits complex spatial and temporal processes. Therefore, urban ecological security could be simulated using CA models.

The CA simulation model used in this paper is based on the concept of GWR. The cellular automata is composed of five basic elements: cell, cell space, neighbor, transformation rules, and time sets. The neighborhood function is the core component of CA’s conversion rules, which embody the essence of CA’s “bottom-up” self-organization evolution. The classical CA algorithms usually employ the traditional global regression statistics such as a logistics regression model to calculate the conversion rules. There is, however, a limitation in using the global regression: the estimated parameters using the global logistics regression model are just mean values of the explanatory variables. The global parameters cannot reflect spatial heterogeneity of the relationships between the dependent variable and the explanatory spatial variables. Based on the first law of geography, near objects are more related than distant objects. Therefore, the explanatory variables may have different degrees of influence on conversion rules for different geographical locations. The conversion rules may not be a trend distribution with a single spatial structure. They may have spatial instability.

To reflect spatial instability in conversion among different land use types, we employed a local regression model—GWR—instead of the global logistics regression model to calculate the conversion rules. GWR is an extension of the traditional multiple linear regression toward a local regression. The regression coefficients of GWR are specific to a location rather than being global estimates [64,65]. The local estimation of the regression coefficients of GWR is expressed by the following equation:

(1) $$y_{i}=\beta_{0}\left(m_{i},n_{i}\right)+\overset{n}{\underset{j=1}{\sum }}\beta_{j}\left(m_{i},n_{i}\right)x_{ij}+\varepsilon_{i}$$

where $\left(m_{i},n_{i}\right)$ is the spatial location of the *i*th observation and $\beta_{j}\left(m_{i},n_{i}\right)$ is the value of the *j*th parameter at point location *i*. The regression parameters of this equation are estimated at each location *i*$\left(m_{i},n_{i}\right)$.

GWR was specifically designed to deal with the spatial non-stationarity of regression coefficients between the dependent variable and explanatory variables by measuring those coefficients locally using local data [66,67,68]. GWR has capability of incorporating various auxiliary variables with spatially varied correlation coefficients. The GWR concept, therefore, may more accurately reflect the characteristics of spatial heterogeneity of ecological dynamic processes. Hence, in the processes of deciding conversion rules, we used GWR instead of global logistics regression to obtain cell conversion probability. We applied the GWR in the CA rules mainly for calculating the probability from one assigned class to other classes. We first used the GWR models to find out relationships between a cell state value and each explanatory variable over space. Based on the relationships we then calculated the conversion probability of each cell to one specified type at each spatial position using the following formula:

(2) $$p_{i}=\frac{e^{y_{i}}}{1.0+e^{y_{i}}}$$

where $p_{i}$ represents the transition probability to one cell state value at position *i*, and $y_{i}$ is the regression coefficient equation for position *i* that was obtained by GWR.

In order to combine with the CA model, it is necessary to give one specific probability for each spatial location. We used the concept of roulette wheel to decide which class would be selected substantially based on the aforementioned calculated probability values. At the same time, we also considered whether or not the selected class values would satisfy the pre-set cell transition probability values.

We forecasted the ecological security status and patterns of 2020 based on the ecological security levels of 2005 and 2010 using the GWR tool in ArcGIS 10.2 software (Environmental Systems Research Institute, Inc., Redlands, CA, USA) [69]. We took the ecological security level as the dependent variable and the indicators calculated in the aforementioned PSR evaluation as the independent variables to obtain the regression coefficients of each variable. Taking simulation of the urban ecological security of 2005–2010 for example, to simulate ecological security status in 2010, we used the number of ecological evaluation classes in 2010 as the termination condition based on the ecological security patterns of 2005. In the process of cell conversion iterations, we first estimated the probability of the currently iterated cell that would turn to each ecological security class. We then determined a class by the method of roulette wheel and the judgement about whether or not the conversion probability of the class satisfied the pre-set cell transition probability. If it satisfied, then the cell would be turned to that class. We stopped the iteration after all of the ecological security classes were satisfied. The total number of iterations was recorded as *N*. For forecasting the urban ecological security of 2020, we took *3N* as the total number of iterations from the ecological security patterns of 2005.

## 3. Results and Discussion

### 3.1. PSR Evaluation

The PSR evaluation results of ecological security values were taken into account of ecological pressure, ecological state, and ecological response. The evaluated ecological security values are distributed in the range of 0–0.7. For a better view, we divided the range into five levels, from Class 1 to Class 5. If a value is less than 0.15, the grid cell will be classified into Class 1. Class 1 means a location with the lowest security. If a value belongs to the range from 0.15 to 0.3, the location will be classified into Class 2. Similarly, Class 3 has values ranging from 0.3 to 0.4. A cell location with Class 3 indicates that it is located at the area with the mid-security level. Class 4 has values ranging from 0.4 to 0.5. The highest level of the urban ecological security is Class 5, which has values greater than 0.5.

#### 3.1.1. Evaluation Results of 2005 and 2010

**Figure 3:**
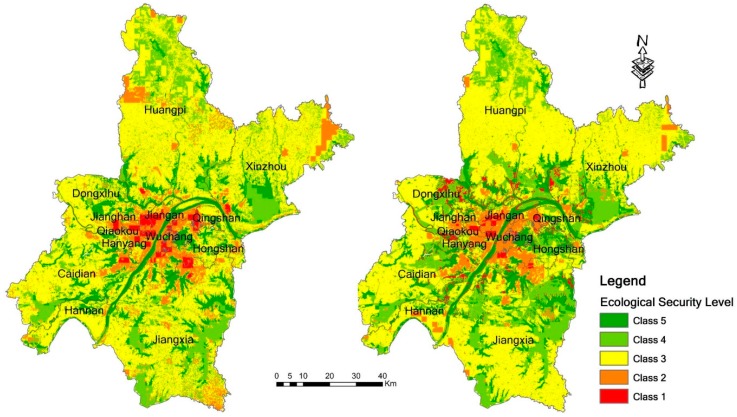
Evaluation results of ecological security in 2005 and 2010 using the PSR model.

Table 2 and Figure 3 show the evaluation results of ecological security in 2005 and 2010. From these results, it can be seen that the ecological security in 2010 has an obvious improvement compared to that in 2005. Class 1 and Class 2 in 2010 represent decreased percentages compared to those in 2005, and there is increase in areas of Class 4 and Class 5 in 2010 compared to 2005.

**Figure 4:**
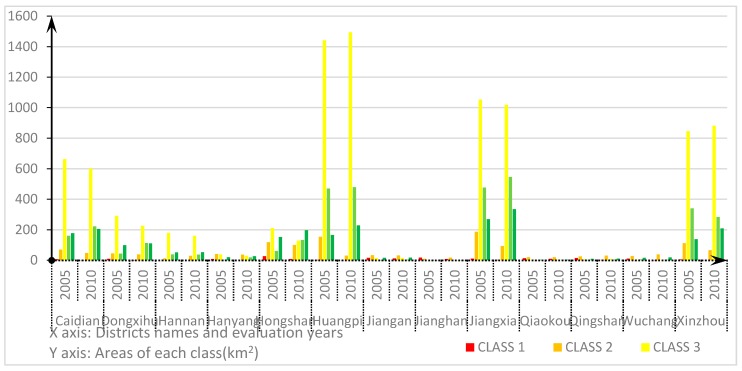
The distributions of ecological security classes in 2005 and 2010.

For the administrative areas, in Figure 4, the ecological conditions in 2005 are similar in the districts of Huangpi, Jiangxia, Xinzhou, Dongxihu, Caidian and Hannan: they all have the largest proportion of Class 3, then followed by Class 4 and Class 5. This indicates good ecological conditions in the administrative areas. Hanyang, Jiangan, Jianghan, Qiaokou, and Qingshan Districts belong to the poor ecological security areas, because of the larger proportions of Class 2 and Class 1. Wuchang District also has a large proportion of Class 2 and Class 1, but the areas belonging to Class 4 and Class 5 in Wuchang are not small. Wuchang District is therefore noted as a district with internal heterogeneity. The areas of Class 1, which indicates the worst ecological security, are mainly distributed in Hongshan, Jianghan, Jiangan, and Qingshan Districts. There have been great changes in overall ecological conditions in 2010 compared to those in 2005. However, the ecological conditions in each administration district have not shown great changes. The ecological conditions in Huangpi, Jiangxia, Xinzhou, Dongxihu, Caidian and Hannan Districts improved. These districts have ecological security levels above the average. Jianghan, Qiaokou, and Qingshan Districts have poor ecological security conditions. The number of the districts with high internal heterogeneity in 2010 increased from one to three districts, including Hanyang, Jiangan, and Wuchang Districts. The areas with the worst ecological security conditions are mainly located in Jiangan, Qiaokou, Jianghan, and Hongshan Districts.

#### 3.1.2. Discussion of the Changes from 2005 to 2010

**Figure 5:**
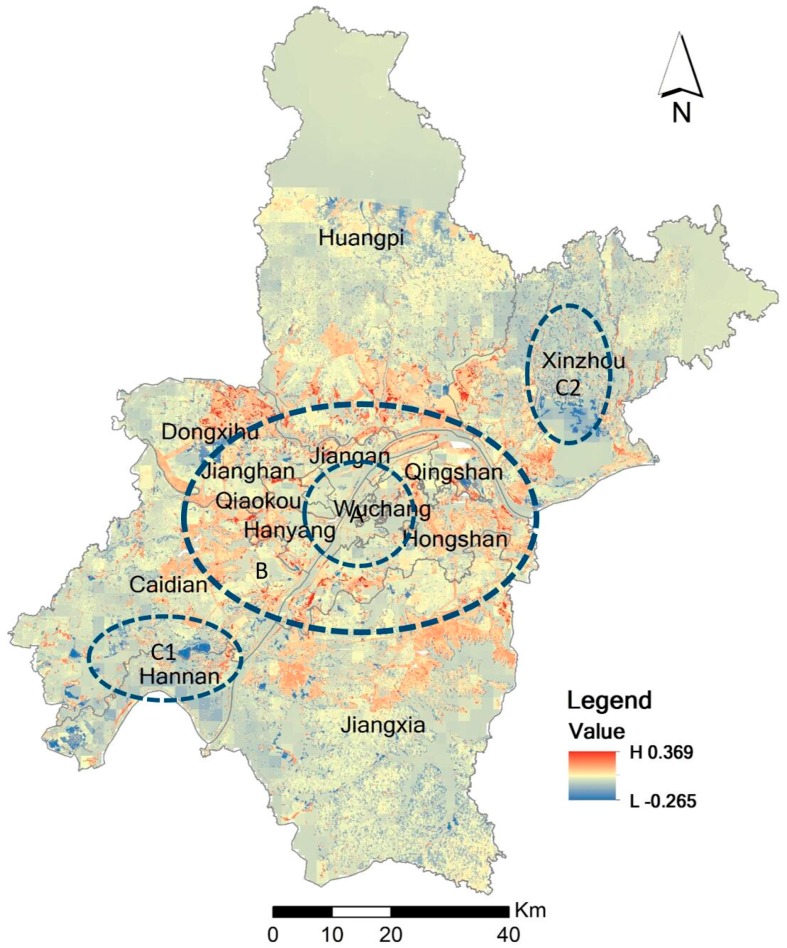
Changes in ecological security from 2005 to 2010.

Figure 5 shows the changes of ecological security from 2005 to 2010. The region with red color means an increase in ecological security level, and the region with blue color means a decrease in ecological security level. From the final ecological security evaluation results of 2005 and 2010, we can see that the ecological environment in the central city has decreased slightly during that five-year period. In the central city (circle A in the figure), the ecological environment remains unchanged or just decreases slightly. From the edge of the central city to the suburban areas, most places have an increased ecological security. In the outskirts of the city, the ecological security situation has shown a certain degree of reduction. This is especially true for Xinzhou district and Hannan Districts (circles C1 and C2).

Most areas in the 13 administrative districts also retain unchanged ecological conditions. For example, more than 80% of the areas in Wuchang and Jianghan Districts maintain their original ecological security levels. Except for Hannan District, the other 12 districts have enhanced conditions as the second largest proportion. In total 16.76% of the areas in the Hannan District have weakened ecological conditions while only 11.41% of areas in that district have enhanced conditions. Hannan District is the only district that has a larger proportion of areas with weakened conditions than areas with enhanced conditions. There are relatively large ecological security-enhanced areas in Jiangxia, Huangpi, Xinzhou, Caidian, Hongshan and Dongxihu Districts. The proportions of enhanced areas in Hongshan and Qingshan Districts are the two highest proportions in all of the districts, at 39.27% and 38.21%, even if their enhanced areas are not as large as those in the previously mentioned districts because of their small total district areas. Except for Hongshan and Qingshan Districts, Hanyang and Dongxihu Districts have enhanced proportions larger than 30%.

**Figure 6:**
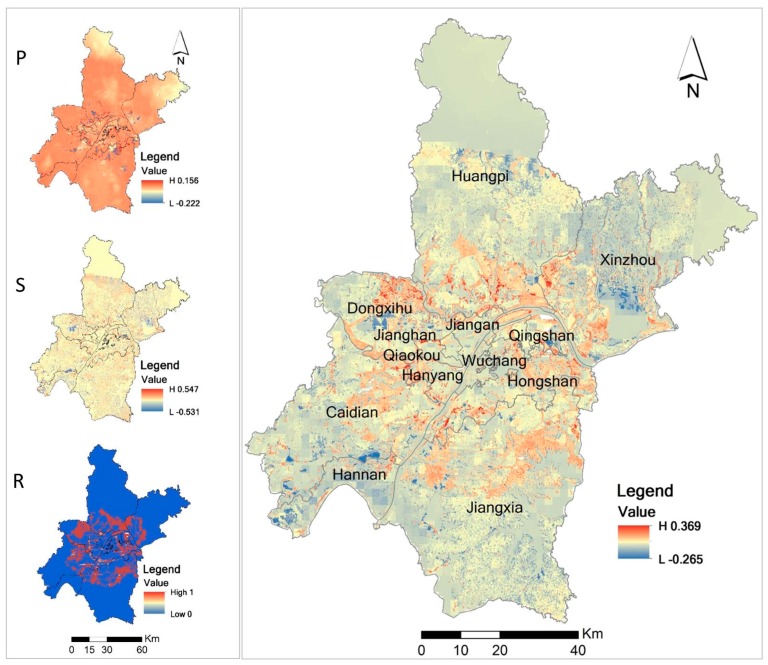
The P/S/R changes and the whole changes of ecological security in Wuhan city from 2005 to 2010.

To find out the reason for ecological security changes from 2005 to 2010 in Wuhan, we analyzed the urban ecological security from the perspectives of ecological pressure (P), ecological state (S), and ecological response (R), respectively. Figure 6 illustrates the P/S/R changes from 2005 to 2010. As shown in Figure 6, the overall ecological environment pressure of Wuhan has significantly increased from 2005 to 2010, and only small areas around the main city have decreased ecological pressure. The ecological pressure in north Huangpi District and northeast Xinzhou District has little change. The areas of ecological improvement are mainly located in the southern part of Huangpi District and some areas in Qingshan, Hongshan, and Dongxihu Districts. The northeastern part of Wuhan city that belongs to the Xinzhou area and the southwest part that belongs to the Hannan District show a weakened ecological environment.

The ecological state basically remained unchanged from 2005 to 2010, and the ecological response was not significant either. There is only a slight decline of the ecological security in the central city. Nevertheless, the construction expansion from the city center to the surrounding areas of the city was significant, which caused most of the surrounding urban areas to have increased ecological environment pressure. The ecological responses in these areas were the most significant. However, the ecological security state of these areas remained basically unchanged or was even slightly enhanced. This is the main ecological security improvement in Wuhan. The ecological response in the suburb areas was small. If combined with urbanization, the ecological security situation remained basically unchanged or slightly decreased from 2005 to 2010.

We used expert knowledge and a literature review to find out the important drivers of ecological security changes in Wuhan. We found that the better ecological planning management and policies greatly accounted for ecological security changes of Wuhan City from 2005 to 2010. The changes in urban planning and land use policies were linked to modifications of landscapes and ecosystems in Wuhan. Good planning, management, and policies played an important role in driving the security changes. These planning management and policies were mainly applied to the outer ring areas around the main urban area, which include Huangpi, Dongxihu, Caidian, Jiangxia, Xinzhou, Hongshan Districts, and the eastern part of Qingshan District. Based on the expert knowledge and literature review we also found that the ecological security environment changes during 2005–2010 resulted from comprehensive multi-factors such as social, political and economic factors. For example, population growth and changing economic conditions were two causes of ecological security change. Urbanization and extended peri-urban settlement threatened various ecosystem processes and drove ecological security change from 2005 to 2010. Rapid economic development and construction efforts in Wuhan led to major ecological security changes from 2005 to 2010. In general, Wuhan has undergone rapid development period in recent years, especially in the central city, where development and construction efforts continued to increase in its main areas. This resulted in a widespread ecological pressure. However, even with the wide and fast development of the city, there was no sharp decline of the ecological state.

### 3.2. The Simulation by the Proposed CA Model

We used the proposed CA model with the GWR concept to simulate the ecological security situation in 2010 based on the ecological security situation in 2005. We then compared and verified the simulation results with the actual ecological security values in 2010. Kappa coefficient statistics were used to measure the simulation results. The calculated kappa coefficient is 0.756, which indicates that the simulation results are good and the proposed CA model can be used to predict future ecological security.

**Figure 7:**
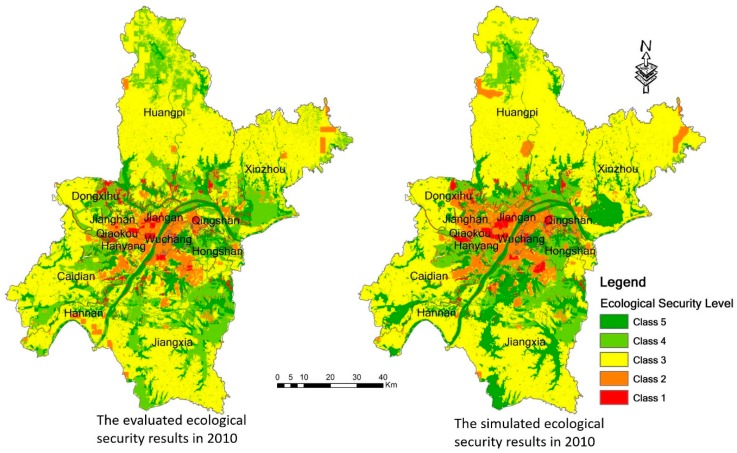
The evaluated ecological security results in 2010 and the simulated ecological security results in 2010.

The evaluated ecological security results in 2010 and the simulated ecological security results in 2010 are shown in Table 3 and Figure 7. From Figure 7, it can be seen that the proportion of each class in the simulated results in 2010 is roughly the same as the actually evaluated results in 2010. From Table 2 it can be seen that the areas with a fair ecological security in both the evaluated results and the simulated results still occupy the largest proportion. However, Class 4 occupies 12.80% in the simulated results and 21.85% in the evaluated results. Class 4 has the largest difference among the five classes. Only the simulated areas of this class are smaller than the actual areas, and the simulated areas of the other four classes are larger than the actual areas.

### 3.3. Prediction Results of 2020

**Figure 8:**
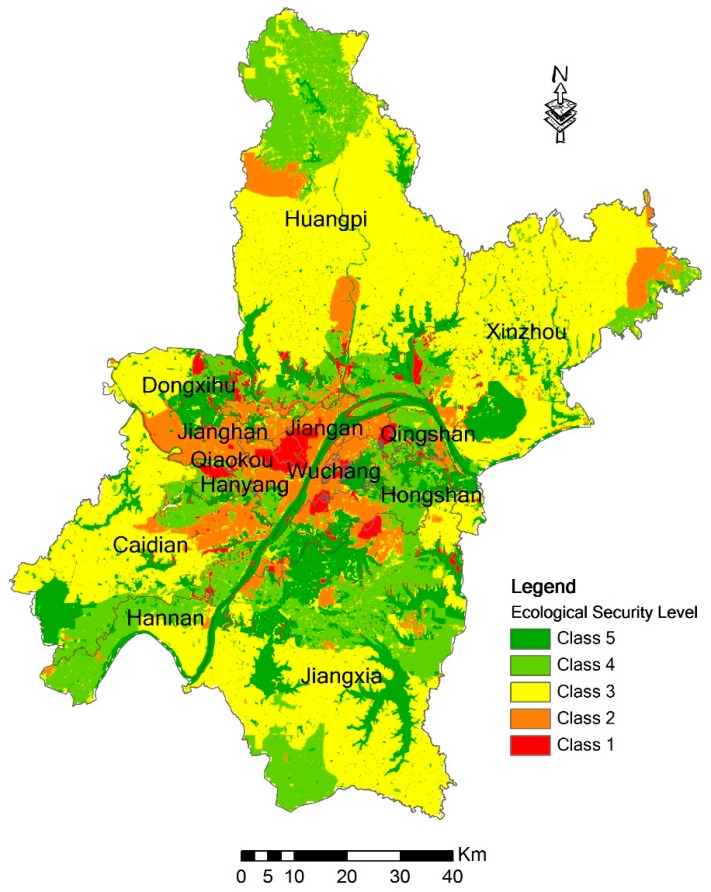
Prediction results of the ecological security in 2020.

We used the proposed CA model to forecast the ecological security situation of Wuhan in 2020. Figure 8 illustrates the predicted results of the ecological security in 2020. As shown in the figure, areas with comparative insecurity (Class 1 and Class 2) are projected to be more concentrated by 2020, especially in Jianghan, Jiangan and Qiaokou Districts. Both of Class 1 and Class 2 occupy more than 70% of the administrative areas in these three districts, and they are the major classes in these three districts. Hanyang District and Wuchang District belong to the areas with a low ecological security, because the proportions with ecological unsafe areas in these two districts are larger than those with secure areas. Caidian, Huangpi, Xinzhou, and Jiangxia Districts belong to the districts with good ecological conditions. These four districts have large areas with mid-security level. The total area with security level higher than the middle level is larger than that with security level lower than the middle level in these four districts. Hannan and Hongshan Districts are the districts with the best ecological conditions in Wuhan because of their large proportions of Class 4 and Class 5. Qingshan District has a big internal difference: the ecological safe areas and unsafe areas account for 42% and 44%, and both are widely distributed. On the contrary, the Dongxihu District has the smallest internal heterogeneity.

**Figure 9:**
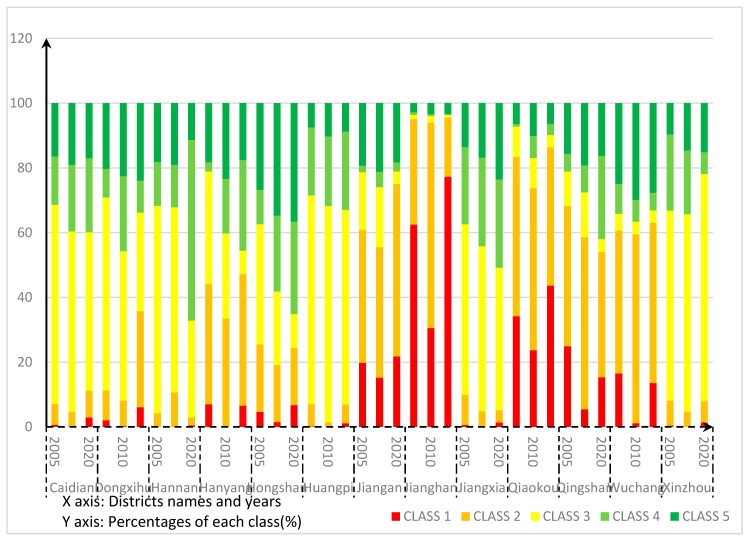
The proportions of each ecological security level in the 13 districts of Wuhan from 2005 to 2020.

Figure 9 illustrates the proportions of each ecological security level in the 13 districts of Wuhan from 2005 to 2020. From the figure, it can be seen that the ecological security situation will have an improvement in the areas of Hannan, Hongshan, Jiangxia, Qingshan, and Jiangan Districts by 2020. Jianghan and Qiaokou Districts should be the areas for future development. These two districts have a lot of areas with low security levels.

## 4. Conclusions

In this paper, we proposed an improved CA approach for simulate and predict urban ecological security at a fine scale (district level). We proposed to first evaluate urban ecological security using the PSR method, and then simulate and predict urban ecological security using the CA model with the GWR concept. We applied the proposed CA approach in a case study—simulating and predicting urban ecological security in Wuhan, China. We first evaluated the ecological security levels in 2005 and 2010 in Wuhan city using the PSR method. We then simulated and predicted the future urban ecological security situations in 2010 and 2020 using the improved CA model with the GWR concept. The results show that our improved CA model is effective and feasible for simulation or prediction of future ecological security.

Although the simulated results are good, there were some limitations in our case study. For example, the Landsat images were used in this case study. The Landsat imagery has a low resolution and many mixed pixels. The mixed pixels made it difficult to accurately classify the satellite images. As the input data for our models, the inaccurate classification results from the satellite images affected the accuracy of our final simulation results. For example, Class 4 generated the largest error in the simulation results of 2010—there is about 9% difference between the simulation results and the evaluated results of Class 4. This has caused the overall simulated ecological security situation lower than the overall actual situation. The other limitation is validation of the CA model. It is impossible to obtain a perfect simulation result using any model. Therefore, it is important to validate the improved CA model for practical applications. In this study, we only used the kappa coefficient indices for validation purpose. However, the Kappa indices have limitations, and sometimes they may be misleading for purposes of accuracy assessment and map comparison [70]. Therefore, we may consider adopting more measures, such as quantity disagreement and allocation disagreement from a cross-tabulation matrix, for validation purposes in the future.

We discussed the reasons for the ecological security changes in Wuhan from 2005 to 2010. The ecological security environment changes between 2005 and 2010 resulted from multiple factors. The ecological security changes were mainly caused by the rapid economic development and construction efforts in Wuhan. Better ecological planning management practices and policies in 2010 also led to the ecological security improvement. These planning management and policies were mainly applied to the outer ring areas around the main urban areas.

Based on the prediction results of 2020, we would like to make the following policy recommendations for each district in Wuhan. For the districts that are continuing to have a comparably high ecological security and have an obvious improvement from 2005 to 2020, such as Hannan, Hongshan, and Jiangxia Districts, the government only needs to maintain the ecological environment. For the districts that will continue to be the concentration areas with the worst ecological security situations of Wuhan city in 2020, such as the Jianghan, Jiangan, and Qiaokou Districts, the government should pay more attention to a centralized development for them. The government should undertake more contiguous land use planning instead of fragmented land use planning in order to make full use of each piece of land. For some districts such as Qingshan District, diverse plans should be made inside the district considering its high internal spatial heterogeneity in 2020.

## Figures and Tables

**Table 1:** PSR evaluation indices and their weights. UGD: urban growth degree; UGC: urban growth capacity; UGP: urban growth pattern; LEF: landscape ecological function; LES: landscape ecological structure; LER: landscape ecological resilience; P: ecological pressure; S: ecological state; R: ecological response.

P-S-R		Criteria		Indices	
Names	Weights	Names	Weights	Names	Weights
Pressure	0.3	UGD	0.5	Proportion of construction land	1.0
		UGC	0.3	Population Density	0.4
				Shortest distances to traffic (road/railway/airport)	0.2 (0.6/0.2/0.2)
				Distances to geometric centers	0.1
				Distances to commercial centers	0.3
		UGP	0.2	Aggregated/linear growth	0.25
				Leapfrog growth	0.75
State	0.5	LEF	0.4	Ecological service values	1.0
		LES	0.4	Shannon diversity	0.2
				Area-weighted plots fractal dimension index	0.4
				Patch density	0.4
		LER	0.2	The fragility index	1.0
Response	0.2	R	1.0	Ecological zoning and environmental plans	1.0

**Table 2:** The evaluation results of ecological security in 2005 and 2010.

	Evaluation Results of 2005		Evaluation Results of 2010	
	Areas (km^2^)	Percentages (%)	Areas (km^2^)	Percentages (%)
CLASS 1	141.90	1.68	47.46	0.56
CLASS 2	854.46	10.09	581.47	6.87
CLASS 3	4753.35	56.11	4568.95	53.98
CLASS 4	1603.92	18.93	1849.09	21.85
CLASS 5	1117.71	13.19	1416.83	16.74

**Table 3:** The simulated and evaluated ecological security results in 2010.

	Simulation Results in 2010		Actual Evaluation Results in 2010	
	Areas (km^2^)	Percentages (%)	Areas (km^2^)	Percentages (%)
CLASS 1	228.86	2.70	47.46	0.56
CLASS 2	694.61	8.21	581.47	6.87
CLASS 3	4850.02	57.30	4568.95	53.98
CLASS 4	1083.58	12.80	1849.09	21.85
CLASS 5	1607.78	18.99	1416.83	16.74
